# Decreased FOXD3 Expression Is Associated with Poor Prognosis in Patients with High-Grade Gliomas

**DOI:** 10.1371/journal.pone.0127976

**Published:** 2015-05-26

**Authors:** Wei Du, Changhe Pang, Dongliang Wang, Qingjun Zhang, Yake Xue, Hongliang Jiao, Lei Zhan, Qian Ma, Xinting Wei

**Affiliations:** 1 Department of Neurosurgery, the First Affiliated Hospital, Zhengzhou University, Zhengzhou 450052, China; 2 Department of Neurosurgery, Peking University People’s Hospital, Beijing, 100044, China; 3 Department of Gastroenterology, the Second Affiliated Hospital of Harbin Medical University, Harbin 150086, China; 4 Prenatal Diagnosis Center, Department of Gynecology and Obstetrics, the First Affiliated Hospital, Zhengzhou University, Zhengzhou 450052, China; The Ohio State University Medical Center, UNITED STATES

## Abstract

**Background:**

The transcription factor forkhead box D3 (FOXD3) plays important roles in the development of neural crest and has been shown to suppress the development of various cancers. However, the expression and its potential biological roles of FOXD3 in high-grade gliomas (HGGs) remain unknown.

**Methods:**

The mRNA and protein expression levels of FOXD3 were examined using real-time quantitative PCR and western blotting in 23 HGG and 13 normal brain samples, respectively. Immunohistochemistry was used to validate the expression FOXD3 protein in 184 HGG cases. The association between FOXD3 expression and the prognosis of HGG patients were analyzed using Kaplan-Meier survival curves and Cox proportional hazards regression models. In addition, we further examined the effects of FOXD3 on the proliferation and serum starvation-induced apoptosis of glioma cells.

**Results:**

In comparison to normal brain tissues, FOXD3 expression was significantly decreased in HGG tissues at both mRNA and protein levels. Immunohistochemistry further validated the expression of FOXD3 in HGG tissues. Moreover, low FOXD3 expression was significantly associated with poor prognosis in HGG patients. Depletion of FOXD3 expression promoted glioma cell proliferation and inhibited serum starvation-induced apoptosis, whereas overexpression of FOXD3 inhibited glioma cell proliferation and promoted serum starvation-induced apoptosis.

**Conclusions:**

Our results indicated that FOXD3 might serve as an independent prognostic biomarker and a potential therapeutic target for HGGs, which warrant further investigation.

## Introduction

Gliomas are the most common primary tumors in the central nervous system (CNS), 70% of which are high-grade gliomas (HGGs), the most frequent and aggressive brain tumors [1]. According to the revised World Health Organization (WHO) classification, HGGs comprise all gliomas with grade III and grade IV (glioblastoma, GBM) [2]. The current standard treatment for HGGs is maximum safe surgical resection followed by adjuvant chemotherapy with DNA alkylating reagent and radiotherapy. Even with this approach, however, the median survival time of patients with GBM and grade III gliomas remains short (only 15 months and 4 years, respectively) [3]. Therefore, there is an urgent need to explored novel biomarkers to improve the diagnosis and treatment of HGGs.

Forkhead box D3 (FOXD3), belonging to the FOX transcription factor family, has been shown to expressed in embryonic stem cells and plays crucial roles in the neural crest development and stem cell biology through specifying the cell lineage [4,5]. In the early mouse embryo, FOXD3 is important for maintaining the pluripotent cells of inner cell mass, neural crest and trophoblast progenitors [6]. Recent studies have reported the roles of FOXD3 in the development of cancer. Dottori et al have demonstrated that ectopic expression of FOXD3 suppresses migration and invasion of melanoma cell lines [7], while FOXD3 silencing in early-migrating neural crest cells leads to an expansion of the melanoblasts [8]. Li et al. have shown that FOXD3 is under-expressed in human neuroblastoma tissues and cell lines, and exhibits tumor suppressor activity by inhibiting neuroblastoma cell proliferation, invasion, metastasis, and reduced growth of xenograft tumors in mice through direct transcriptional regulation of NDRG1 [9]. In gastric cancer, the expression of FOXD3 is down-regulated mainly due to the hypermethylation of promoter and FOXD3 expression is correlated with survival time of patients [10]. Furthermore, FOXD3 significantly inhibits the proliferation and invasion of gastric cancer cell lines [12]. However, the role of FOXD3 in HGGs has not been described.

In the present study, we examined the expression of FOXD3 in HGGs and analyzed its association with the prognosis of patients. Moreover, we explored the effects of FOXD3 expression on proliferation and apoptosis of glioma cells in vitro.

## Materials and Methods

### Patients and Tissue Specimens

A total of 184 HGG tissues were obtained from patients undergoing surgery treatment at the Department of Neurosurgery, Peking University People's Hospital between November of 2005 and May of 2012. This cohort of patients included 42 cases of grade IV and 142 cases of grade III gliomas. The study population consisted of 69 women and 115 men (mean age, 55 years; age range, 18–78 years). All tumor tissues were obtained from the initial surgery, and none of the patients had received anticancer therapy before tumor excision. The histologic subtypes and pathologic grades of all glioma samples were confirmed by two pathologist (blind to the data for the patient). For comparisons, 13 normal brain tissue samples were obtained from patients with brain trauma undergoing urgent decompressive operation in the same hospital. Overall survival (OS) was defined as the period between surgery and death of glioma or the last follow-up. Progression-free survival (PFS) was defined as the time from initial surgical to tumor progression in MRI or death from glioma. The present study was approved by the Ethic Committee of Peking University People’s Hospital, and written informed consent was obtained from each patient. All specimens were handled and made anonymous according to the ethical and legal standards.

### Cell lines and culture conditions

Five malignant human glioma cell lines, LN405, U118, SW1080, T98M and U87MG were purchased from the American Type Culture Collection (ATCC, USA) and maintained in Dulbecco’s modified Eagle medium (DMEM) supplemented with 10% fetal bovine serum (FBS). All cells were incubated at 37°C in a humidified chamber containing 5% CO_2_. The cells were harvested in the logarithmic phase of growth for use in the experiments outlined below.

### RNA extraction and real-time quantitative PCR

Total RNA was isolated with TRIzol reagent (Invitrogen, USA) according to the manufacturer’s instructions. The reverse transcription was performed using Transcriptor First Strand cDNA Synthesis Kit (Roche, Indianapolis, IN). Real-time PCR was performed with SYBR Green PCR Master Mix (Applied Biosystems, Foster City, CA) on a Bio-Rad CFX96 real-time PCR system. The expression of FOXD3 was calibrated to that of GAPDH using the 2^-ΔCt^ method. The primers using in the amplification were as follows: FOXD3 forward: 5’-GACGACGGGCTGGAAGAGAA-3’, reverse: 5’-GCCTCCTTGGGCAATGTCA-3’; and GAPDH forward: 5’- CTCCTCCTGTTCGACAGTCAGC-3’, reverse: 5’- CCCAATACGACCAAATCCGTT-3’.

### Western blotting

Tissues and cell lines were resuspended in RIPA lysis buffer containing protease inhibitor cocktail (Amresco, Solon, OH, USA). 20 μg of total protein was separated by 10% sodium dodecyl sulfate polyacrylamide gel electrophoresis (SDS-PAGE) and transferred onto a polyvinylidene fluoride (PVDF) membrane (0.45 mm, Millipore, Bedford, MA, USA). After blocking non-specific binding sites for 60 min with 8% non-fat milk, membranes were incubated overnight at 4°C with primary antibodies against Cleaved Caspase-3 (1:1,000; Millipore, Billerica, MA, USA), FOXD3 (1:1,000; Abcam, USA) or GAPDH (1:1,000; Cell Signaling Technology, USA). After four washes, membranes were probed with HRP-conjugated secondary antibodies and bands were detected with enhanced chemiluminescence reagent (Amersham Bioscience, Piscataway, NJ, USA). Band density of FOXD3 was measured with ImageJ software (National Institutes of Health, Bethesda, MD) and was standardized to that of GAPDH. A calibrator protein sample from a same normal brain tissue was loaded in each western blotting run and the relative protein expression level was calibrated by this sample.

### Immunohistochemistry

The tumor samples were fixed in 4% neutral formalin, embedded in paraffin and cut into 4-μm-thick sections. The sections were deparaffinized in xylene and rehydrated with graded alcohol, and 3% H_2_O_2_ was applied to block the endogenous peroxide activity for 15 min at room temperature. The antigen was retrieved at 95°C in 0.01 M sodium citrate buffer (pH 6.0) for 20 min, and the sections were blocked with normal goat serum. The slides were incubated with rabbit anti-FOXD3 antibody (1:100; Abcam, USA) at 4°C overnight. After washing, the sections were incubated with horse reddish peroxidase (HRP)-labeled anti-rabbit secondary antibody (Santa Cruz, USA) for 1 hour at room temperature. The antigen-antibody complexes were visualized using diaminobenzidine (DAB) and counterstained with haematoxylin.

The specimens were analyzed by two indepepdent pathologists who were blinded to the patients’ clinical outcomes. Discrepancies between the observers were found in less than 10% of the examined slides, and a consensus was reached after further review. The total FOXD3 immunostaining score was calculated as the proportion of positive tumor cells and the intensity score. Briefly, The proportion of tumor cells was scored as: 0 (no positive staining), 1 (1%-9% positive staining), 2 (10%–50% positive staining), and 3 (51%-100% positive staining). The intensity of staining was graded as: 0 (no staining), 1 (pale yellow), 2 (bright yellow), and 3 (dark brown). The total immunostaining score was calculated with the value of percent positivity score × staining intensity score. For each batch experiment, a positive control slide from the same tissue block was examined and the final immunostating score was calibrated by the positive control. Slides with ≥ the median staining index score were classified as having “high expression”, whereas < the median staining index score were classified as “low expression”.

### siRNA, Plasmid and transfection

Expression of FOXD3 was knocked down using siRNA duplexes as the following sequence: negative control (NC) 5’-UGGUUUACAUGUCGACUAA-3’, FOXD3 5’-ACGACGGGCUGGAAGAGAA-3’. The full-length FOXD3 coding sequence was cloned into pcDNA3.1(+) vector (Invitrogen, Carlsbad, CA). All constructs were fully sequenced. The siRNA duplexes and expression vector were transfected into SW1080 and U87MG using Lipofectamine 2000 (Invitrogen, USA), respectively.

### 3-(4,5-dimethylthiazol-2-yl)-2,5-diphenyl tetrazolium bromide (MTT) assay

Cells were trypsinized 24 hours after transfection and seeded into 96-well plates. At 1–5 day after cell inoculation, 50 μl of MTT solution (1 mg/ml) was added into each well and incubated for 4 hours, and then the medium was replaced by 200 μl of dimethyl sulfoxide (DMSO) (Sigma Aldrich, St Louis, MO). After shaking at room temperature for 10 min, the absorbance of each well at 490 nm was detected using a micro-plate auto-reader (Bio-Rad, Richmond, CA, USA).

### 5-ethynyl-2′-deoxyuridine (EdU) retention assay

An EdU assay was performed using the Cell Light EdU DNA imaging kit (Guangzhou RiboBio Co., Ltd., Guangzhou China) to measure cell proliferation. 48 hours after transfection, cells were seeded in 96-well plates and exposed to 25 mM EdU for 2 h at 37°C, and were then fixed in 4% paraformaldehyde. Following permeabilization with 0.5% Triton X-100, the 16 Apollo reaction cocktail (Guangzhou RiboBio Co., Ltd., Guangzhou China) was added and the cells were incubated for 30 min. Subsequently, the DNA of the cells was stained with 4',6-diamidino-2-phenylindole (DAPI) (Guangzhou RiboBio Co., Ltd., Guangzhou China) for 15 min and visualized under a fluorescent microscope (IX81; Olympus Corporation, Tokyo, Japan). The cell count was analyzed.

### Apoptosis assay

48 h after transfection, the cells were incubated in serum-free medium for another 48 h. The cells were then harvested and resuspended in 500 μl of binding buffer. 5 μl of annexin V-FITC solution and 10 μl PI (1 μg/ml) were added to these cells for 30 min away from the light. Apoptosis were detected on a flow cytometer (Becton Dickinson, USA) according to the manufacturer’s instructions.

### Statistical analysis

Statistical analyses were performed with SPSS 17 software (SPSS Inc., Chicago, IL, USA). The age of patients was converted into categorical variable at the median (55 years). KPS score was converted into categorical variable at 70 because patients with KPS < 70 have remarkably worse prognosis as previously reported [12]. The immunostaining score was converted into categorical variable at the median (6) of all cases. Chi-square test was used to analyze the relationships between FOXD3 expression level and clinicopathological parameters. Kaplan-Meier survival curves were plotted and compared with the log-rank test. Hazard ratio (HR) and 95% confidence intervals (CI) were calculated by Univariate and multivariate Cox proportional hazards regression models to assess the effects of clinicopathological parameters and FOXD3 expression level on the survival of patients. Mann-Whitney test was used to compare FOXD3 expression and EdU data between two groups. Two-tailed *P* < 0.05 was considered to be statistically significant.

## Results

### FOXD3 expression was downregulated in HGGs

**Fig 1:**
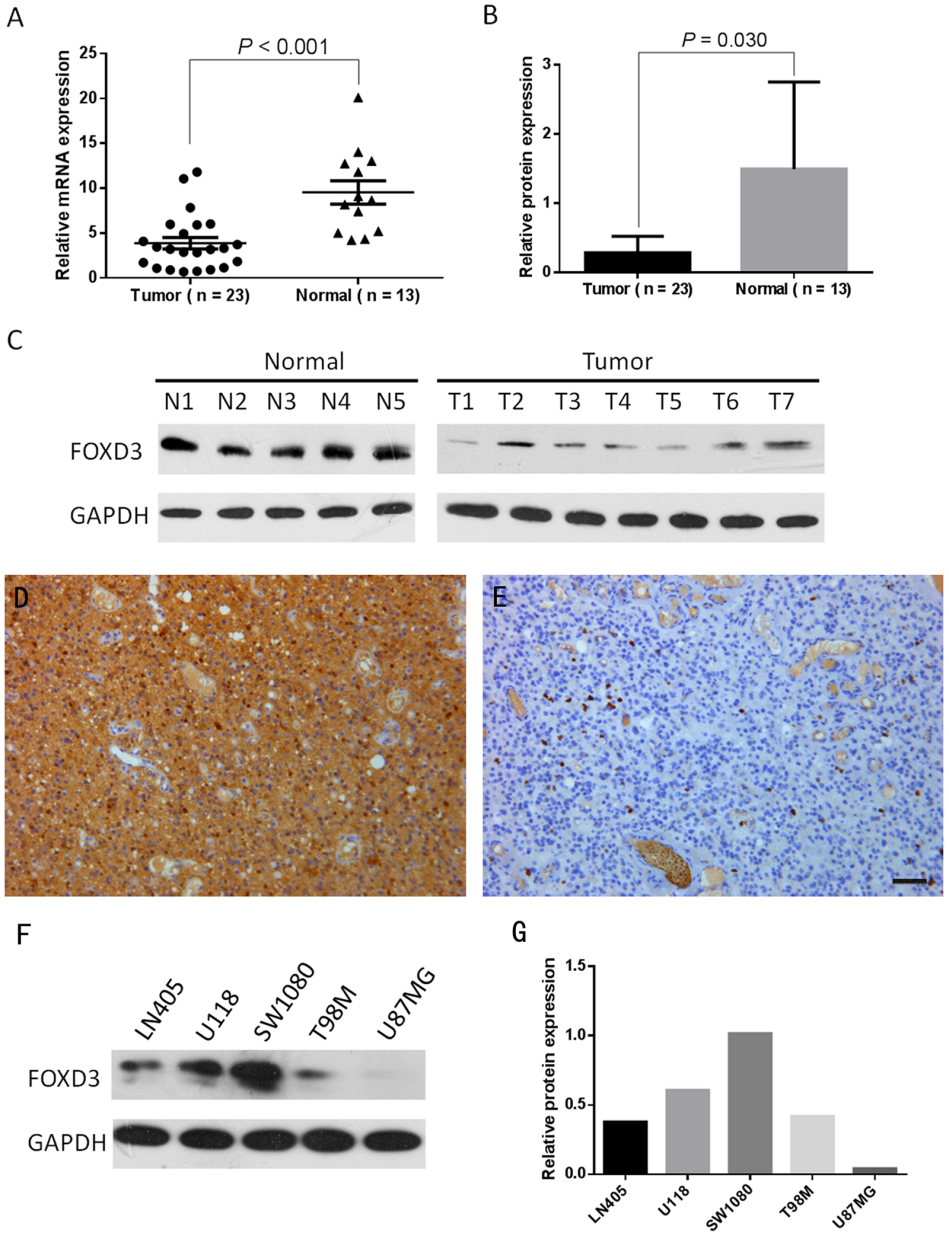
FOXD3 expression in HGG tissues and glioma cell lines. A: FOXD3 mRNA expression in 23 HGG cases and 13 control tissues. B: FOXD3 protein expression in 23 HGG cases and 13 control tissues. C: Representative protein expression of FOXD3 in 7 HGG cases and 5 control tissues by western blotting. D and E: Representative immunohistochemical staining of high expression and low FOXD3 expression in HGG cases, respectively. F and G: FOXD3 expression in five glioma cell lines by western blotting. Scale bar, 50μm.

First, we assessed FOXD3 expression in 23 fresh HGG specimens and 13 normal brain tissues using real-time quantitative PCR and western blotting. The results revealed FOXD3 mRNA expression was significantly decreased in glioma tissues in comparison to normal brain (*P*<0.001, Fig 1A). Western blotting analysis further showed that glioma had lower FOXD3 protein expression than normal brain (*P* = 0.030, Fig 1B and 1C). Immunohistochemistry showed FOXD3 protein was mainly expressed in the nuclear of glioma and normal brain cells. (Fig 1D and 1E).

Furthermore, we examined the protein expression levels of FOXD3 in five glioma cell lines including LN405, U118, SW1080, T98M and U87MG cells. SW1080 cells exhibited higher FOXD3 expression than other cells, while U87MG exhibited complete loss of FOXD3 expression (Fig 1F and 1G). Thus, we chose SW1080 and U87MG cell lines to further investigate.

### Downregulated FOXD3 expression was correlated with poor prognosis in HGG patients

**Fig 2:**
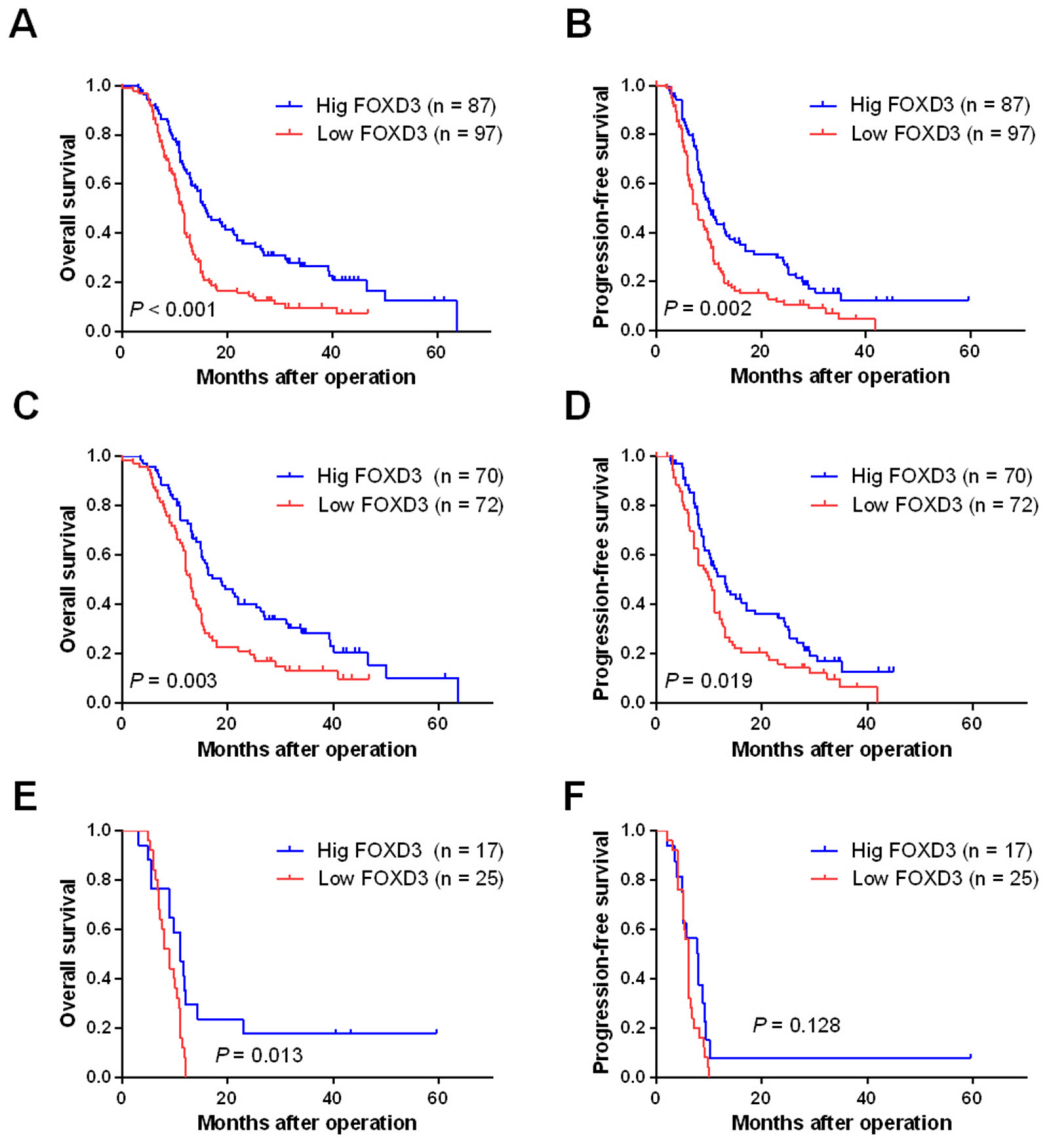
Low FOXD3 expression was assotiated with poor prognosis in HGG patients. A and B: Kaplan-Meier curves OS and PFS in all HGG patients according to the expression levels of FOXD3, respectively. C and D: Kaplan-Meier curves OS and PFS in grade III glioma patients according to the expression levels of FOXD3, respectively. E and F: Kaplan-Meier curves OS and PFS in grade IV glioma patients according to the expression levels of FOXD3, respectively.

Correlations between FOXD3 expression and clinical characteristics were analyzed using the chi-square test. As shown in Table 1, there were no statistical associations between FOXD3 expression and the clinical parameters, such as age, gender, location, resection degree, KPS or WHO grade (III and IV) (*P* > 0.05). We further explored the prognostic value of FOXD3 in glioma. As shown in Fig 2A and 2B, HGG patients with low FOXD3 expression showed significantly worse OS and PFS than those with high FOXD3 expression (*P*<0.001 and *P* = 0.002, respectively). Univariate (Table 2) and multivariate (Table 3) analyses showed that FOXD3 expression level was an independent prognostic factor for both OS and PFS (*P* < 0.001 for both) of HCC patients. Nevertheless, neither immunostaining intensity nor positive proportion of FOXD3 alone was significantly associated with the prognosis of patients (data not shown). Moreover, our stratified analysis showed that high FOXD3 expression was significantly with better OS in patients with either WHO grade III or grade IV diseases (Fig 2C and 2E). However, high FOXD3 expression was significantly with better PFS in patients with WHO grade III diseases (Fig 2D) but not grade IV gliomas (Fig 2F), probably due to the limited sample size of this subpopulation.

**Table 1:** Relationship between FOXD3 expression and clinicopathological features of 184 patients with HGGs.

Variables		Total	FOXD3 expression		*P*-value
			Low	High	
Gender					.620
	Female	69	38	31	
	Male	115	59	56	
Age(years)					.090
	< 55	79	36	43	
	> = 55	105	61	44	
Location					.530
	Frontal lobe	67	31	36	
	Parietal lobe	16	12	4	
	Occipital lobe	5	2	3	
	Temporal lobe	55	29	26	
	Insular lobe	2	1	1	
	Thalamus	10	7	3	
	Cerebellum	9	5	4	
	Brain stem	1	1	0	
	Lateral ventricles	10	5	5	
	Fourth ventricle	2	0	2	
	Other site	7	4	3	
WHO grade					.315
	III	142	72	70	
	IV	42	25	17	
Resection degree					.840
	GTR	149	78	71	
	NTR	35	19	16	
KPS score					0.72
	< 70	38	21	17	
	> = 70	146	76	70	

Abbreviations: GTR, gross total removal; KPS score, Karnofsky performance score; NTR, near total removal.

**Table 2:** Univariate analysis analysis of factors associated with survival and progression in HGG patients.

Variables	OS			PFS		
	HR	95% CI	*P*-value	HR	95% CI	*P*-value
FOXD3 expression (high/low)	0.535	0.387–0.741	0.000	0.614	0.447–0.844	0.003
Gender (male/female)	0.902	0.652–1.247	0.532	0.912	0.662–1.255	0.571
Age (> = 55/<55)	1.951	1.398–2.723	0.000	1.944	1.397–2.703	0.000
WHO grade (IV/III)	2.345	1.613–3.410	0.000	3.019	0.050–4.445	0.000
KPS score (> = 70/<70)	1.326	1.041–1.689	0.043	1.302	0.975–1.739	0.063
Resection degree (NTR/GTR)	1.578	1.113–2.237	0.013	1.641	1.235–2.180	0.008

Abbreviations: CI, confidence interval; HR, Hazard ratio; OS, overall survival; PFS, progression free survival.

**Table 3:** Multivariate analysis of factors associated with survival and progression in HGG patients.

Variables	OS			PFS		
	HR	95% CI	*P*-value	HR	95% CI	*P*-value
FOXD3 expression (high/low)	0.539	0.387–0.753	0.000	0.659	0.477–0.910	0.011
Gender (male/female)	0.914	0.659–1.267	0.589	0.925	0.672–1.274	0.634
Age (> = 55/<55)	1.776	1.268–2.485	0.001	1.844	1.317–2.580	0.000
WHO grade (IV/III)	2.459	1.675–3.609	0.000	3.040	2.052–4.504	0.000
KPS score (> = 70/<70)	1.244	1.013–1.528	0.047	1.241	0.958–1.608	0.102
Resection degree (NTR/GTR)	1.407	1.015–1.950	0.021	1.583	1.156–2.168	0.011

### FOXD3 silencing promoted glioma cell proliferation and inhibited serum starvation-induced apoptosis

**Fig 3:**
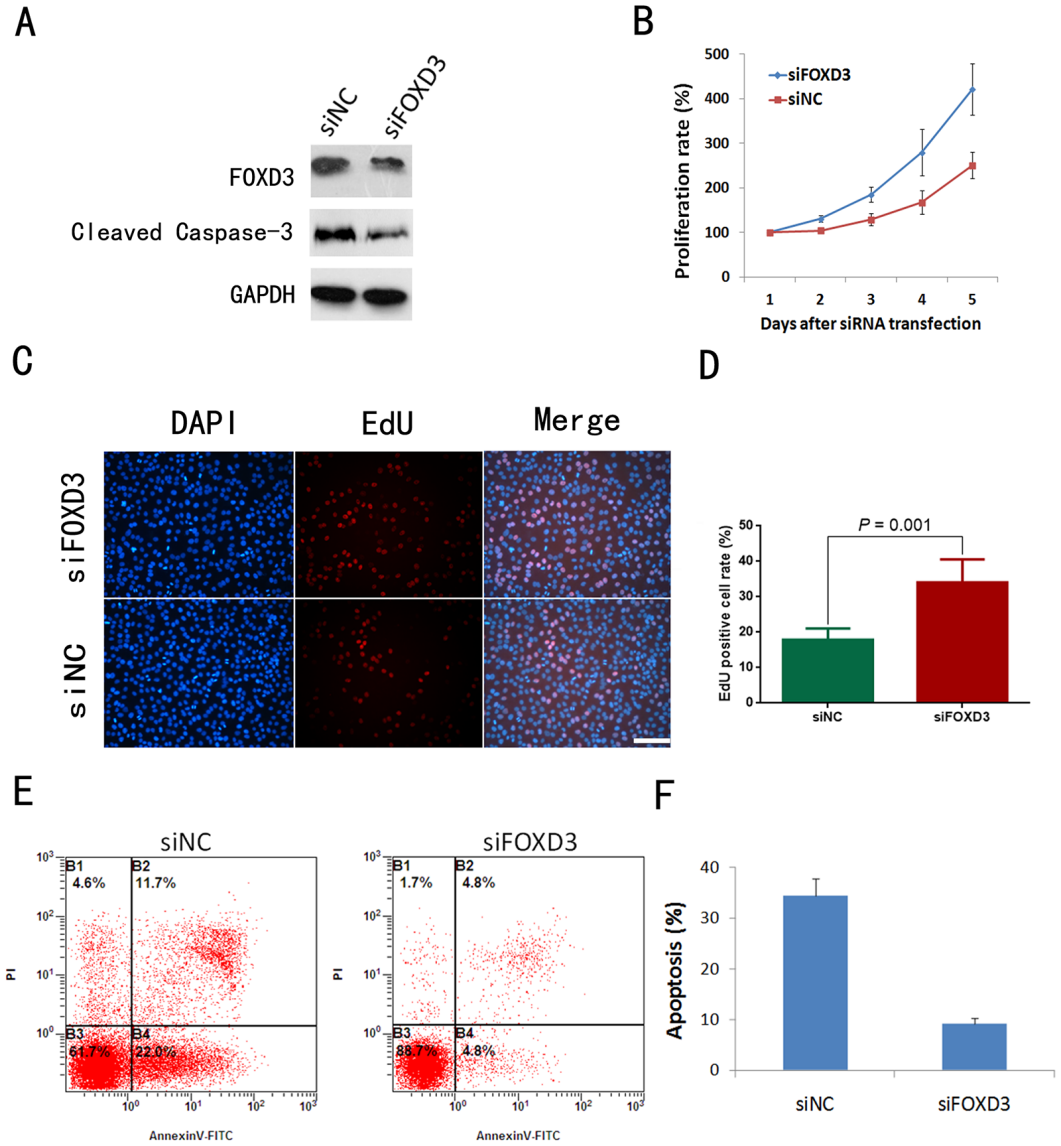
FOXD3 silencing promoted proliferation and inhibited starvation-induced apoptosis in SW1080 cells. A: Western blotting analysis of FOXD3 and cleaved caspase-3 expression in SW1080 cells. B: Cell proliferation assay showing significantly enhanced proliferation rate of FOXD3-silenced SW1080 cells in comparison with siNC-treated SW1080 cells (*P*<0.05) by MTT assay. C: Representative images of EdU assay in SW1080 cells. D: Statistical results from five independent EdU assays. E: Representative results of apoptosis assay by flow cytometry in serum-starvated SW1080 cells. F: Statistical results from three independent apoptosis assays. Summarized results are expressed as mean ±SD. EV, empy vector; NC, negative control. Scale bar, 50μm.

To explore the function of FOXD3 in gloma, siRNA transfection was employed to knockdown FOXD3 expression in SW1080 cells. Western blotting assays showed that the protein expressions of FOXD3 in FOXD3 siRNA-transfected SW1080 cells (siFOXD3) were reduced than negative control (NC) cells (Fig 3A). MTT assay revealed that FOXD3 silencing expression remarkably promoted the proliferation of the SW1080 cells (Fig 3B). EdU assay showed that silencing FOXD3 expression significantly increased the proportion of proliferating SW1080 cells (Fig 3C and 3D). To further investigate the effects of FOXD3, apoptosis was analyzed by flow cytometry analysis. Cells were cultured in serum-free medium for 48 hours. Annexin V-FITC/PI dual staining analysis showed that the downregulation of FOXD3 inhibited apoptosis in siRNA-transfected SW1080 cells (Fig 3E and 3F). Western blotting analysis showed that silencing of FOXD3 remarkably suppressed the cleavage of pro-casepase 3 (Fig 3A).

### Overexpression of FOXD3 inhibited glioma cell growth and enhanced serum starvation-induced apoptosis

**Fig 4:**
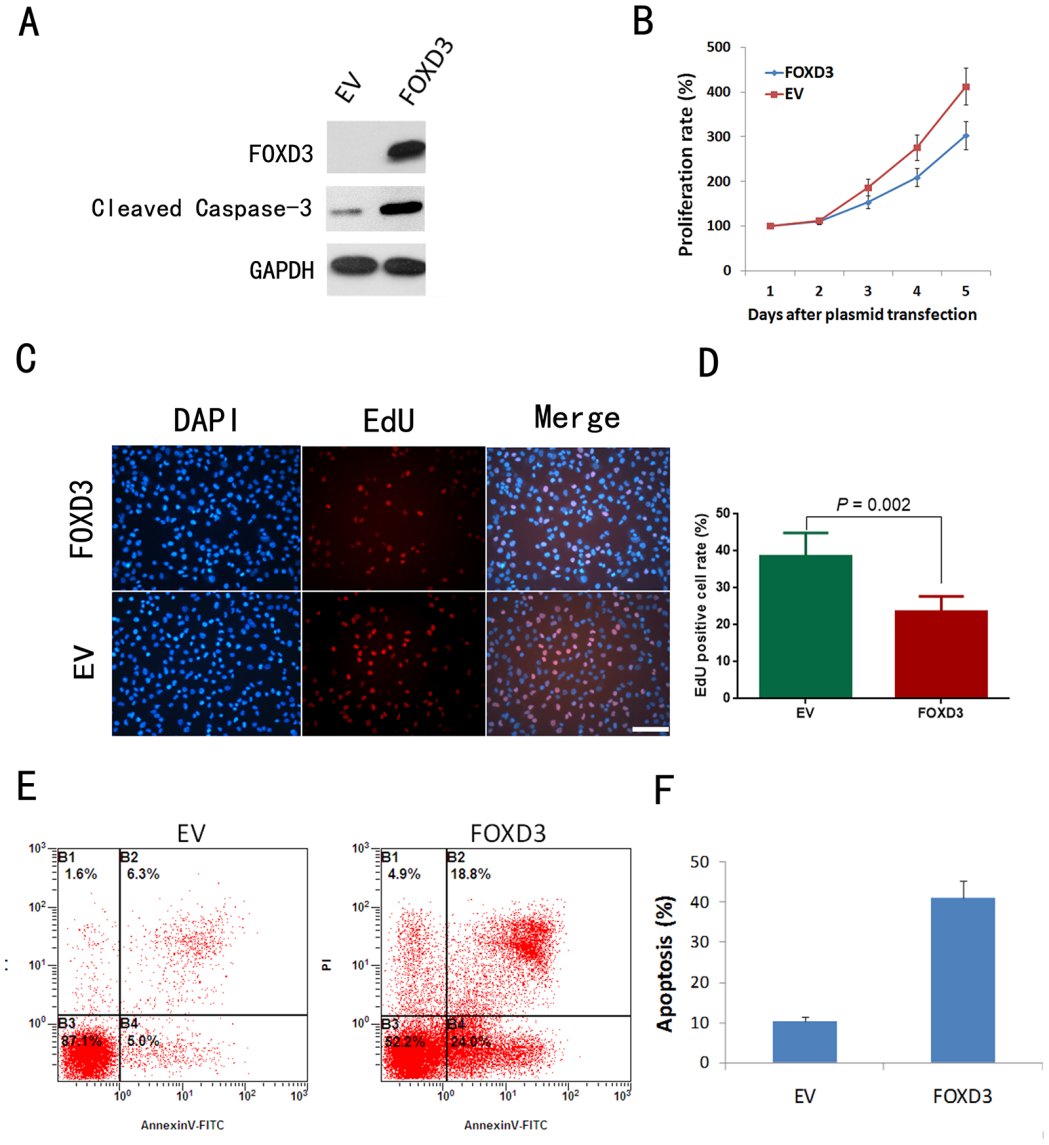
FOXD3 overexpression suppressed proliferation and enhanced starvation-induced apoptosis in U87MG cells. A: Western blotting analysis of FOXD3 and cleaved caspase-3 expression in U87MG cells. B: Cell proliferation assay showing significantly enhanced proliferation rate of FOXD3-overexpressed U87MG cells in comparison with EV-treated U87MG cells (*P*<0.05) by MTT assay. C: Representative images of EdU assay in U87MG cells. D: Statistical results from five independent EdU assays. E: Representative results of apoptosis assay by flow cytometry in serum-starvated U87MG cells. F: Statistical results from three independent apoptosis assays. Summarized results are expressed as mean ±SD. EV, empy vector; NC, negative control. Scale bar, 50μm.

Next, FOXD3 cDNA was cloned into pcDNA3.1(+) expression vector and transfected into U87MG cells. Empty vector (EV) was used as negative control. Western blotting analysis showed an increased level of FOXD3 expression in FOXD3-transfected U87MG cells (Fig 4A). MTT analysis indicated that FOXD3-transfected cells grew much slower than the control cells (Fig 4B). EdU assay showed that FOXD3 overexpression significantly decreased the proportion of proliferating U87MG cells (Fig 4C and 4D). Next, we performed apoptosis assay to evaluate the effects of FOXD3 on the apoptosis of cells cultured in serum-free medium for 48 hours. Flow cytometry analysis showed that the upregulation of FOXD3 enhanced apoptosis in U87MG cells (Fig 4E and 4F). Western blotting analysis showed that FOXD3 overexpression remarkably promoted the cleavage of pro-casepase 3 (Fig 4A).

## Discussion

In the present study, we examined the expression of FOXD3 in HGGs and found that FOXD3 expression was significantly decreased in HGGs in comparison to normal brain at both mRNA and protein levels. Moreover, decreased FOXD3 expression was significantly associated with poor prognosis of patients. In addition, our in vitro analyses showed that FOXD3 inhibited the proliferation of glioma cells and promoted starvation-induced cell apoptosis. Taken together, these results indicated that decreased FOXD3 might play an important role in the development of HGGs.

As an evolutionarily conserved transcriptional regulator, FOXD3 plays important roles in biological processes, such as differentiation, proliferation, apoptosis and tumorigenesis [13,14]. Many studies have demonstrated FOXD3 to be a tumor suppressor in various cancer cells [15]. Genome-wide location analysis has identified many proapoptotic genes as the potential transcriptional targets of FOXD3 in mice and humans gastric cancer [10]. Both over-expression and knockdown of FOXD3 studies have demonstrated that FOXD3 significantly inhibits the proliferation, metastasis and invasion of breast and gastric cancer cells [10,11,16,17]. In line with these findings, we found that FOXD3 promoted serum starvation-induced apoptosis and inhibited proliferation in glioma cells, suggesting that loss of FOXD3 might play an important role in the development of glioma.

Since FOXD3 exerts inhibiting effects on the initiation and progression of cancer, it is plausible that FOXD3 expression is downregulated in a range of malignancies, including breast, colorectal cancers as well as neuroblastoma, melanoma and chronic lymphocytic leukemia (CLL) [11,16,18–21]. Moreover, Li et al have demonstrated that FOXD3 expression is downregulated in neuroblastoma and FOXD3 overexpression significantly promotes the growth, metatstasis and angiogenesis of neuroblastoma [9]. Similarly, we demonstrated a decrease of FOXD3 expression in HGGs and overexpression of FOXD3 significantly may inhibit the proliferation of glioma cells partly by promoting cell apoptosis under stress. Collectively, these data suggest that FOXD3 may exert its tumor suppressing effects through different mechanisms in different types of malignancies.

The causes of FOXD3 downregulation in cancer cells is still not clear. Several studies have shown that epigenetic inactivation of FOXD3 by promoter methylation during the development of CLL, colorectal and gastric cancer [10,18,20,21]. Nevertheless, whether promoter methylation accounts for the downregulation of FOXD3 in HGGs needs further investigation.

The molecular mechanisms by which FOXD3 inhibits the growth of glioma remain unknown. Abel et al have demonstrated that FOXD3 induces cell cycle arrest via p53-dependent pathway in melanoma [22]. Moreover, Katiyar et al have reported that FOXD3 inhibits the migration and invasion of melanoma cells through promoting the transcription of Rho family GTPase 3 [19]. Li et al recently have demonstrated that FOXD3 suppresses the growth of neuroblastoma through promoting the transcription of NDRG1 [9], which also serves as a tumor suppressor in glioma [23,24]. However, the biological roles of the interaction between FOXD3 and NDRG1 in HGGs need further investigation in future.

With regard to its tumor suppressor role, it is not surprising that FOXD3 expression level may predict the prognosis of cancer patients. Chu et al have indicated that down-regulation of FOXD3 is associated with poor prognosis in breast cancer patients [16]. Cheng et al have reported that gastric patients with FOXD3 promoter methylation have shorter survival than those without methylation [10]. Similarly, Li et al have shown that high FOXD3 expression is associated with good prognosis in neuroblastoma patients [9]. In line with these data, our study also demonstrated that low FOXD3 expression was associated with poor OS and PFS in HGG patients, suggesting that FOXD3 expression level may serve as an independent prognostic biomarker for HGGs, which need further validation in larger prospective patient population in future.

There were several limitations in our study. First, there was more grade III than grade IV gliomas in our study. The small sample size of grade IV cases limited the statistical power in our stratified analysis. Therefore, the biological roles and clinical significance of FOXD3 expression in grade IV gliomas need further investigation in larger population. Second, the sample size was relatively small and the major histopathological type of grade III glioma was anaplastic astrocytoma (111 cases) in our study, which limited the power of our stratified analyses according to the subtypes of glioma. Therefore, the fidings of our study need further validation in larger population in future.

In conclusion, our data suggested that FOXD3 expression was down-regulated in HGGs and decreased FOXD3 expression was an indicator of unfavorable prognosis in HGG patients. Moreover, in vitro studies demonstrated that FOXD3 inhibited proliferation and enhanced starvation-induced apoptosis in glioma cells. Our findings provide evidence that FOXD3 might serve as a novel prognostic biomarker and a potential therapeutic target for HGGs, which warrant further investigation.
